# Altering the viscoelastic properties of mucus-grown *Pseudomonas aeruginosa* biofilms affects antibiotic susceptibility

**DOI:** 10.1016/j.bioflm.2023.100104

**Published:** 2023-01-21

**Authors:** Kaitlyn R. Rouillard, Matthew R. Markovetz, William J. Kissner, William L. Boone, Lucas M. Plott, David B. Hill

**Affiliations:** a Marsico Lung Institute, The University of North Carolina at Chapel Hill, Chapel Hill, NC, 27599, USA; b Joint Department of Biomedical Engineering, North Carolina State University and the University of North Carolina, Chapel Hill, NC, 27599, USA; c Department of Physics and Astronomy, The University of North Carolina at Chapel Hill, Chapel Hill, NC, 27599, USA

**Keywords:** *Pseudomonas aeruginosa*, Biofilm, Mucus, Respiratory infection, Viscoelasticity, Antibiotic resistance

## Abstract

The viscoelastic properties of biofilms are correlated with their susceptibility to mechanical and chemical stress, and the airway environment in muco-obstructive pulmonary diseases (MOPD) facilitates robust biofilm formation. Hyperconcentrated, viscoelastic mucus promotes chronic inflammation and infection, resulting in increased mucin and DNA concentrations. The viscoelastic properties of biofilms are regulated by biopolymers, including polysaccharides and DNA, and influence responses to antibiotics and phagocytosis. We hypothesize that targeted modulation of biofilm rheology will compromise structural integrity and increase antibiotic susceptibility and mucociliary transport. We evaluate biofilm rheology on the macro, micro, and nano scale as a function of treatment with a reducing agent, a biopolymer, and/or tobramycin to define the relationship between the viscoelastic properties of biofilms and susceptibility. Disruption of the biofilm architecture is associated with altered macroscopic and microscopic moduli, rapid vector permeability, increased antibiotic susceptibility, and improved mucociliary transport, suggesting that biofilm modulating therapeutics will improve the treatment of chronic respiratory infections in MOPD.

## Highlights

• Biofilms grown in pathological human airway mucus exhibit viscoelastic behavior that is dominated by the elastic modulus.
• Molecules smaller than 20 nm in diameter diffuse with water but inert dextrans are actively transported through the biofilm, decreasing residence time.
• Tobramycin treatment alone decreases biofilm viability but does not significantly reduce the viscoelastic properties of biofilms.
• Biofilm disruption with a biopolymer potentiates the efficacy of tobramycin, decreases it's viscoelastic properties, and increases mucociliary transport.

## Graphical abstract

**Figure:**
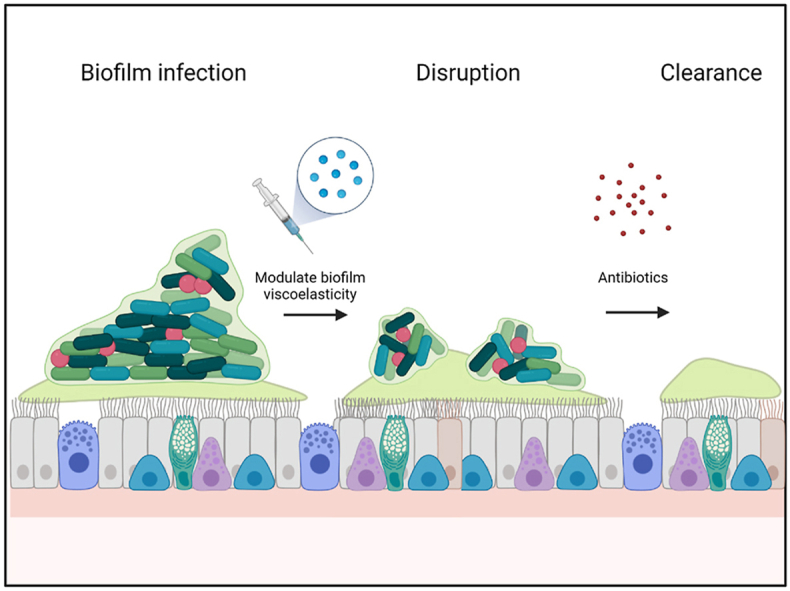

## 1 Introduction

Muco-obstructive pulmonary diseases (MOPD) including cystic fibrosis (CF) and chronic obstructive pulmonary disease (COPD) are characterized by chronic inflammation and progressive obstruction and degradation of the airways [1,2]. Pathological mucus is characterized by increased viscoelastic properties (storage, G’, and loss, G”, moduli, complex viscosity, η*), and adhesion to the underlying epithelium that likely disrupts normal mucociliary clearance (MCC) and promotes a chronic inflammatory response [[3], [4], [5], [6]]. Mucus stasis also facilitates colonization of the airway by opportunistic pathogens like *Pseudomonas aeruginosa.* [7,8] Chronic infection occurs in the form of bacterial biofilms, which are aggregates of bacteria protected by a matrix of extracellular polymeric substances (EPS) [[8], [9], [10]]. The composition of the EPS is influenced by multiple factors including bacteria species and the growth environment [[9], [10], [11]]. This matrix of polysaccharides, lipids, proteins, and DNA affords mechanical strength to the biofilm and offers protection from mechanical and chemical challenges [9,12,13]. Mechanical challenges in the airway include ciliary beating and phagocytosis [[14], [15], [16], [17]]. Chemical challenges include antibiotics and reactive oxygen and nitrogen species (ROS/RNS) [16,18]. Biofilms are able to modulate the composition of the EPS in response to these challenges, resulting in difficult to eradicate infections and rising antibiotic resistance [11,17,19].

The physical structure of the biofilm decreases antibiotic efficacy, contributing to chronic infection and antibiotic resistance [10,12,16,20]. Thus, methods of disrupting the biofilm to improve eradication have been extensively investigated. For example, alginate oligosaccharides have been shown to disrupt bacterial biofilms and potentiate antibiotic action [[21], [22], [23]]. Enzymatic DNA degradation via DNase also decreases the viscoelastic properties of biofilms and increases antibiotic susceptibility [[24], [25], [26]]. Nitric oxide-releasing chitosan oligosaccharides were shown to simultaneously decrease the viscoelastic properties of biofilms and viability [[27], [28], [29]]. Altogether, these studies indicate that both cationic and anionic polysaccharides may be capable of disrupting biofilm mechanics to improve eradication from the disease state airway [27,29]. We therefore posit that disruption of the biofilm architecture, either increasing or decreasing the viscoelastic moduli, will be associated with increased susceptibility to antibiotics.

We have previously reported on the growth of *P. aeruginosa* biofilms in human bronchial epithelial (HBE) mucus, which is biophysically and biochemically similar to human airway mucus [10,30,31]. We determined that mucus concentration was the dominant factor for biofilm viscoelastic properties, with increased mucus concentration correlating with greater biofilm viscoelastic moduli and resistance chemical challenge [10]. Herein, we describe the evaluation of biofilms grown in mucus at 4% solids, which is relevant for moderate airway disease [30]. Biofilm rheology is evaluated on three length scales: macro, micro, and nano to characterize bulk rheology, polymer network behavior and heterogeneity, and the low viscosity fluid that fills the space within the polymer mesh, respectively, as a function of treatment [3,32]. The bulk viscoelastic properties of biofilms affect mucociliary transport (MCT) and phagocytosis [3,32], biofilm microrheology describes microscale spatial heterogeneity as well as the polymer network [[32], [33], [34]]; and nanorheology quantifies vector permeability and fluid movement [3,35]. A systematic evaluation of biofilm rheology on these length scales is essential for the design of efficacious antibiofilm strategies that will increase the likelihood of permanent eradication.

## 2 Methods

*Mucus preparation and biofilm growth.* Human bronchial epithelial (HBE) cells were procured from the Marsico Lung Institute Tissue Procurement Center at UNC Chapel Hill and cultured in MilliCell inserts with custom air-liquid interface media [3,30]. The cultures were maintained at 37 °C with 5% CO_2_ for four weeks until the cells differentiated, developed cilia, and began producing mucus [3,30]. The cultures were washed with PBS and culture washing were collected, pooled, dialyzed, and concentrated as previously described to prepare HBE mucus at a high concentration of solids, which was determined via dry weight measurements [3,30]. Mucus was diluted with sterile PBS to 4% solids and kept at −80 °C until use.

A 3 mL overnight culture of *P. aeruginosa* strain PAO1 (ATCC# 47085) was prepared from frozen stock and reconstituted in fresh tryptic soy broth (TSB, Difco). The bacteria solution was incubated with shaking at 37 °C for ∼4 h until reaching an optical density at 600 nm of 0.25, which corresponds to 10^8^ CFU/mL [13]. Bacteria were diluted 100-fold into HBE mucus and rotated for 10 min to mix. Mucus with bacteria (total volume 75 μL) was added to a 96-well plate. Empty wells were filled with 200 μL sterile MilliQ water to prevent evaporation. The well plate was incubated with shaking (100 rpm) at 37 °C for 24 h until a biofilm formed. The biofilm is a turbid, viscous aggregate that is easily removed from the well with a positive pressure pipette [13,36]. The 50 μL biofilm was removed from the well and added to a microcentrifuge tube for treatment or analysis.

*Rheology*. Macrorheology was performed with cone and plate rheometry using a Discovery Hybrid Rheometer-3 (TA Instruments) equipped with a 20 mm 1° cone at a gap of 30 μm, fill volume of 45 μL [3]. The linear viscoelastic regime (LVR) was determined using a strain sweep (0.01–10% strain) at 0.5 Hz and 5 Hz. Macroscopic moduli were determined via frequency sweeps (0.1–10 Hz) at a strain within the LVR (1% strain) and quantified at 1 Hz. Biofilms were evaluated in technical pentaplicate from three separately grown batches of biofilms. Microrheology was measured by particle tracking microrheology (PTMR) was performed using 0.1% w/v yellow-green, fluorescent carboxylated or PEGylated microspheres (0.5 and 1 μm, Invitrogen) and fluorescence microscopy with a Nikon TE2000 microscope using a 40x air objective and Grasshopper camera GS3 [33]. Mucus samples were beaded with a 1:600 dilution of microspheres and allowed to rotate at 4 °C overnight prior to analysis. Biofilms were beaded with a 1:600 dilution of microspheres added to mucus with the planktonic bacteria prior to incubation. A 7 μL sample of mucus or biofilm was added to a window made from parafilm on a glass microscope slide and sealed with a glass coverslip, as previously described [10,13]. Videos were recorded, and bead movement was tracked with custom Python GUI with the TrackPy package (https://doi.org/10.5281/zenodo.34028) as a backend. Microscopic moduli were calculated using custom Matlab (© 2022 The MathWorks, Natick, MA) scripts which have been published previously [3,33,37]. An extensive description of Gaussian Mixture Modeling can be found in Markovetz et al., 2022 [37]. Three slides per biofilm were prepared and ten videos acquired per slide. Each biofilm condition was prepared in three separately grown batches of biofilms [13].

*Microsphere modification and characterization.* Carboxylated microspheres (Invitrogen) with diameters of 1.0 and 0.5 μm were modified with PEG using carbodiimide chemistry as previously described [38]. Briefly, N-Ethyl-N′-(3-(dimethylamino)propyl)carbodiimide hydrochloride (EDC, 20 mg) and N-Hydroxysuccinimide (NHS, 10 mg) were added to 100 μL microspheres and 900 μL PBS pH 6.5 and allowed to activate with rotation at room temperature for 15 min. Then, 50 mg of 2 kDa methoxy-PEG-amine (Creative PEGWorks) was added and allowed to react for 4 h. Modified particles were collected via centrifugation and washed thrice with PBS pH 7.4. Stock and modified particles were characterized for diameter and ζ-potential using a Malvern ZetaSizer Nano diluted 1:1000 in PBS pH 7.4. Particles were diluted 1:600 into mucus for incorporation into the biofilm.

*1-dimensional migration assay.* Nanorheology of biofilms was characterized via nanoparticle diffusion in one direction through a glass capillary [[39], [40], [41]]. Biofilms (40 μL) were loaded into 1 mm (VitroCom Cat# 8100–050) capillary tubes with 1 μL fluorescent tracers at one end: 3–5 kDa FITC-dextran (Sigma), 70 kDa FITC-dextran (Sigma), >30 kDa FITC-poly-l-lysine (Sigma), or 20 nm yellow-green fluorescent carboxylated nanospheres (Invitrogen). The tubes were sealed on both ends with a 1:1:1 mixture of Vaseline, lanolin, and paraffin (Valap) and placed into a custom-built 3D-printed 384-well plate wherein columns were etched to hold the capillary tubes. The well plate was placed into a Tecan fluorometer maintained at 37 °C and fluorescent signal was measured every 15 min for 24 h. Fluorescence from the tracer particles was recorded in a 384-well plate format, representing 4 mm of diffusion per well. The fluorescence along the capillary was fitted with Gaussian curve and the Stokes-Einstein relationship was applied to determine the biofilm viscosity [40,41]. Biofilms were evaluated in biological duplicate from three separately grown batches of biofilms. The diffusion coefficient is determined by fitting a Gaussian to the intensity pattern of the fluorescent molecule throughout the tube using:

I(x)=I0e(x−x0)22Dt

where *I* is the measured intensity as a function of distance along the capillary tube, *x*. *D* is the diffusion coefficient and *x*_*0*_ is the position of the fluorescence peak within the capillary tube. Once the diffusion coefficient was measured, the effective viscosity was determined by the Stokes-Einstein relationships, specifically:

$$\eta_{eff}=\frac{k_{B}*T}{D*6\pi *r}$$

where *k*_*B*_ is the Boltzmann constant, *T* is temperature, and *r* is probe radius. The hydrodynamic radius, r_h_, of each probe was calculated using a molecular weight to r_h_ converter: https://www.fluidic.com/toolkit/hydrodynamic-radius-converter.

*Biofilm exposure to tobramycin, TCEP, PK and HA*. Biofilms were treated with tobramycin (Sigma) and tris-(2-carboxyethyl)phosphine (TCEP, Sigma), >30 kDa poly-l-Lysine, (PK, Sigma), or hyaluronic acid (HA, Lotioncrafters ELMW 80–110 kDa CAS No. 9067-32-7) individually and in combination. The tobramycin concentrations were 0, 10, 100, and 1000 μg/mL. The TCEP concentration used was 10 mM. The concentrations of PK and HA were 10, 100, and 1000 μg/mL. Biofilms were injected with 5 μL, total, of test agent(s) dissolved in PBS. Exposures lasted for 24 h at 37 °C, and viability and rheology were evaluated immediately after. To determine viability, biofilms were added to 450 μL sterile MilliQ, disrupted via pipetting, serially diluted, and plated on tryptic soy agar (TSA, Difco). Colony counts were quantified after 24 h incubation at 37 °C. Each exposure condition was repeated in three separately grown and evaluated batches of biofilms.

*Confocal laser scanning microscopy.* Biofilms were imaged as a function of treatment with a Leica Stellaris5 confocal laser scanning microscope (CLSM). Staining was performed with propidium iodide (PI, 1 μL of 1 mg/mL ThermoFisher), and BacLight Green (1 μL of stock, ThermoFisher). Live cells are thus stained green and DNA, both environmental and from dead cells, is red. Each biofilm was imaged in ten separate locations for quantification of red and green pixels.

*Mucociliary transport*. Mucus, native biofilms, and treated biofilms were added to transporting racetrack HBE cultures termed mucociliary transport devices (MCTD) which have been previously described [42]. Fluorescent microspheres (1 μm yellow green carboxy) were tracked using fluorescence video microscopy, and MCT and cilia beat frequency (CBF) were quantified using custom Python and Matlab scripts. Mucociliary transport is presented in a swarmchart of all microsphere velocities and the median MCT rate. Cilia beat frequency is presented as the mean ± standard deviation of the mean for three separate measurements of each culture and treatment. Each biofilm was evaluated in duplicate in cross exposed MCTDs.

**Statistical analysis.** Biofilms were evaluated from at least three separately grown batches of biofilms. Statistical significance was determined using a two-tailed Student's *t*-test with Bonferroni correction for viability, cone and plate rheometry, 1-DMA assays, and cilia beat frequency measurements. The Kolmogorov-Smirnov test was applied to microrheology experiments.

**Data availability.** All data and scripts are available upon request.

## 3 Graphical methods

**Figure:**
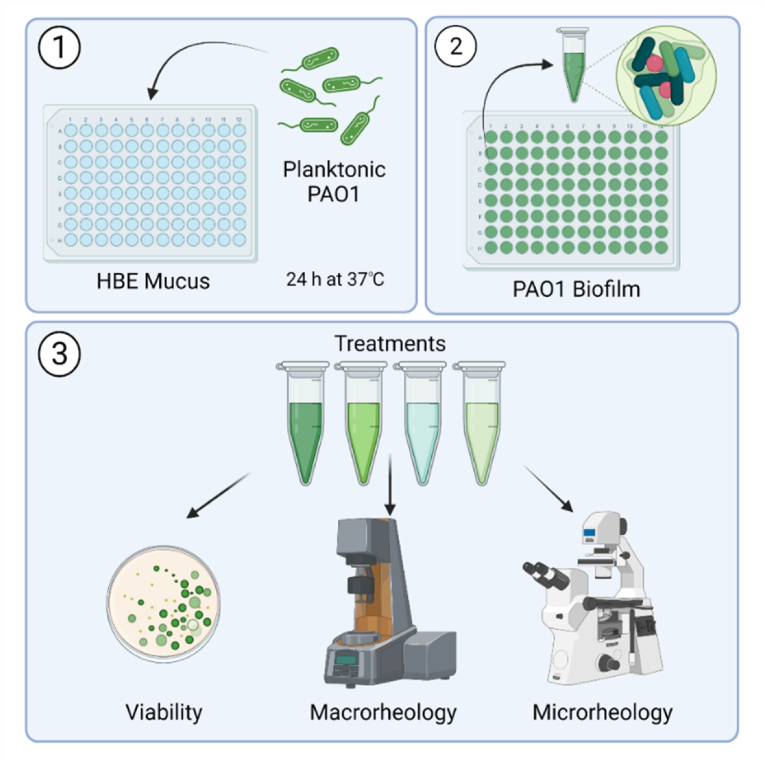

**Graphical methods:** PAO1 biofilms were grown in HBE mucus for 24 h at 37 °C with shaking at 100 rpm. Biofilms were isolated from the well plate and treated with tobramycin, TCEP, poly-l-lysine, or hyaluronic acid individually or in combination. Viability was assessed with colony counting, macrorheology was performed with cone and plate rheometry, and microrheology was performed with fluorescence video microscopy. Created with BioRender.com.

## 4 Results

### 4.1 Biofilms behave as viscoelastic solids, dominated by the elastic modulus

**Fig. 1:**
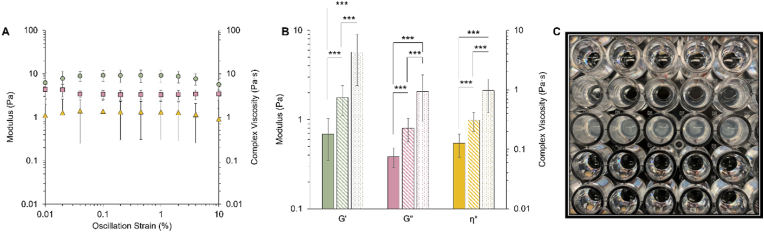
Macrorheology of *P. aeruginosa* biofilms measured via cone and plate rheometry. A) The linear viscoelastic regime (LVR) of *P. aeruginosa* biofilms wherein the elastic modulus (G′, green circle), the viscous modulus (G″, pink square) and complex viscosity (η*, yellow triangle) behave linearly regardless of strain. B) Macroscopic moduli quantified at 1 Hz for 4% mucus (solid), 4% mucus with planktonic PAO1 (hashed), and PAO1 biofilms in 4% mucus (dots). Data is presented as the mean ± standard deviation of five technical replicates in biological duplicates from three separately grown batches of biofilms (n = 30) ***p < 0.001 C) Representative image of biofilms that form in HBE mucus. (For interpretation of the references to colour in this figure legend, the reader is referred to the Web version of this article.)

*Pseudomonas aeruginosa* biofilms were grown in 4% (w/w) mucus for 24 h and demonstrated elastic modulus-dominated behavior, in agreement with previous work [10]. The strain sweep (Fig. 1A) shows the linear viscoelastic regime (LVR) wherein the viscoelastic properties (G’, G”, and η*) of biofilms behave linearly. Macroscopic moduli were evaluated at 1 Hz at a strain within the LVR (1%) as 1 Hz is representative of tidal breath [3,13,30] (Fig. 1B). The elastic modulus dominates within the LVR, indicating that the biofilm behaves as a viscoelastic solid [10,32]. For reference, uninfected 4% mucus as well as 4% mucus with planktonic PAO1 are also dominated by the elastic modulus with a value on the same order of magnitude as measured in these biofilms (Fig. 1B) [10]. Thus, the addition of planktonic bacteria increases (p < 0.001) macroscopic moduli which are increased to an even greater extent (p < 0.001) after biofilm growth.

**Fig. 2:**
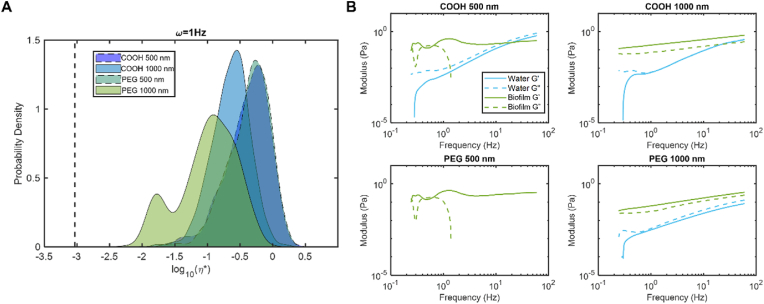
Microrheology of *P. aeruginosa* biofilms measured via particle tracking microrheology at 1 Hz. A) Probability density of biofilm complex viscosity as a function of bead surface chemistry and diameter. Dashed vertical line indicates the viscosity of water. B) Elastic (G′, solid) and viscous (G″, dashed) moduli of biofilms measured as a function of bead surface chemistry and diameter. Data is representative of every tracked bead in biological duplicates from three separately grown batches of biofilms (n > 1000).

Biofilm microrheology was also dominated by the elastic modulus, but the measured η* values varied slightly with bead size and surface chemistry (Fig. 2). The 0.5 μm beads, both COOH and PEG, exhibited similar η* values for PAO1 biofilms. Of these microrheology experiments, only the 0.5 μm beads measured statistically similar η* distributions (p = 0.201) as determined by the Kolmogorov-Smirnov test. The 1 μm COOH beads measured similar η* values as the 0.5 μm beads. However, 1 μm PEG beads exhibited a bimodal distribution of biofilm η* values, with a more solid-like component on par with the η* value measured by the other bead types and a more liquid-like component that is closer to the viscosity of water. These data suggest that larger, PEGylated beads do not intercalate within the biofilms as effectively as the COOH and 0.5 μm PEG beads, and that the 1 μm PEG beads may be excluded to the exterior more water-like part of the biofilm matrix. Thus, the remainder of PTMR experiments were performed with 1 μm COOH beads which measured a uniform η* for biofilms and are the beads traditionally used in our PTMR experiments [3,33].

**Fig. 3:**
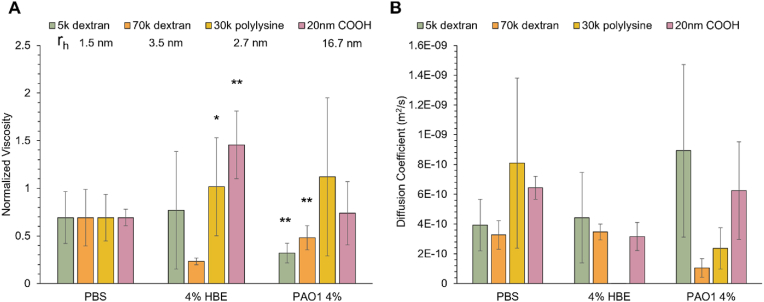
Nanorheology of *P. aeruginosa* biofilms measured via the 1-dimensional migration assay. A) Normalized biofilm dynamic viscosity as a function of probe with each probe hydrodynamic radius (r_h_). B) Measured diffusion coefficient for each probe in PBS, 4% HBE mucus, or PAO1 biofilms grown in 4% mucus. Data is presented as the mean ± standard deviation of biological duplicates from three separately grown batches of biofilms (n > 6). Statistical significance was determined with a two-tailed Student's *t*-test with Bonferroni correction compared to the probe in PBS.

The 1-directional migration assay (1-DMA) was used to probe the mesh network of PAO1 biofilms grown in 4% mucus. Because diffusion occurs in one direction, the fluorescence signal can be evaluated as a function of distance traveled and fit with a Gaussian curve to determine the diffusion coefficient. As the probes are sufficiently small, <200 nm, their diffusion through mucus and biofilms can be characterized as simple Brownian motion [43]. Two sizes of FITC-labeled dextrans (5 kDa and 70 kDa), FITC-labeled poly-l-lysine (30 kDa), and 20 nm COOH beads were used to evaluate vector permeability of mucus and the biofilm (Fig. 3). Viscosity data were normalized to probe diffusion in PBS to mitigate thermal noise. Uniquely, both sizes of dextrans diffused more effectively through PAO1 biofilms than in PBS. As probe diffusion through the biofilm is normalized to its diffusion in PBS, these data suggest the presence of some form of transport different from diffusion. Individual active or passive motion of bacteria within a biofilm can occur [44]. It is possible that motion of the individual bacteria within the biofilm may facilitate more rapid diffusion of the nanometer sized dextrans that do not interact with the mucus mesh. Previous reports have shown that fluid is actively transported through biofilms at rates ranging of 1–10 μm/s for *Bacillus subtilis* [45] and up to 66 μm/s in *Pseudomonas aeruginosa* [46]. Using the Stokes-Einstein relations, we can calculate that a 70 kDa dextran molecule will diffuse roughly 10 μm/s, indicating that active transport, even over small distance within the biofilms, may impact our results. Active transport would cause the probes to move more rapidly and further than they would under Brownian diffusion, which our analysis will interpret as a lower viscosity. Elucidating the exact cause of the rapid motion of the 70 kDa dextran probes in biofilms will be the focus of future research.

In contrast, the poly-l-lysine, which is a cationic biopolymer, exhibited significant heterogeneity in biofilm permeation, which is likely due to chemical interactions between the positively charged biopolymer and the negatively charged components of the biofilm matrix, slowing diffusion. The largest probe utilized, the 20 nm COOH beads, diffused through biofilms at a similar rate as in water, suggesting they are smaller than the mesh size of the biofilm polymer matrix [3]. The diffusion coefficients of each probe measured in PBS (Fig. 3B) are in agreement with theoretical values and support the validation of this assay.

### 4.2 Biofilm disruption increases antibiotic efficacy

**Fig. 4:**
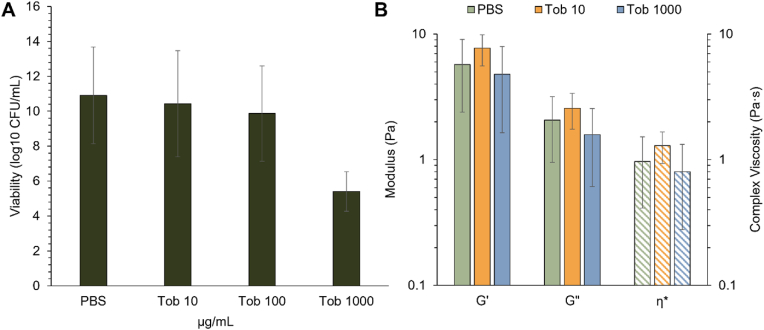
Effect of tobramycin treatment on *P. aeruginosa* biofilms. A) Biofilm viability. B) Viscoelastic moduli of *P. aeruginosa* biofilms after treatment with PBS (green) tobramycin 10 μg/mL (orange) or 1000 μg/mL (blue) as measured via cone-and-plate rheometry at 1Hz. Data is presented as the mean ± standard deviation of biological duplicates from three separately grown batches of biofilms (n = 6). Statistical significance was determined with a two-tailed Student's *t*-test. (For interpretation of the references to colour in this figure legend, the reader is referred to the Web version of this article.)

**Fig. 5:**
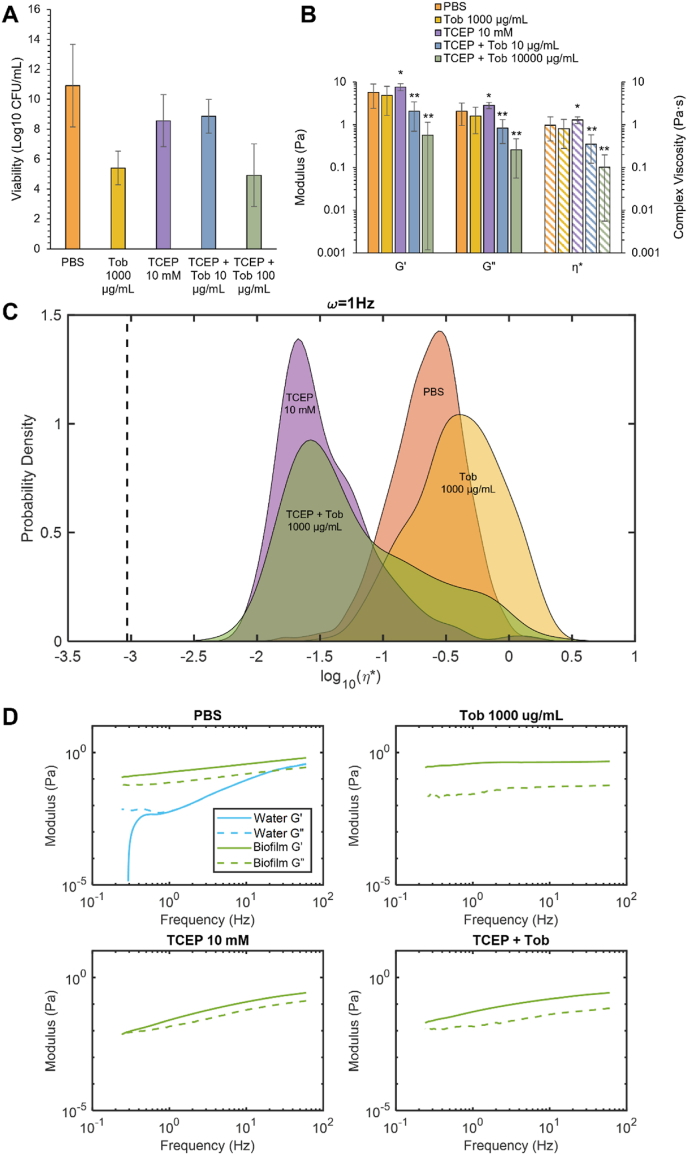
Viscoelastic properties of *P. aeruginosa* biofilms after treatment with TCEP and tobramycin. PBS (orange); Tob 1000 μg/mL (yellow); TCEP 10 mM (purple); TCEP + Tob 10 μg/mL (blue); TCEP + Tob 1000 μg/mL (green). A) Biofilm viability after treatment with PBS, Tob, TCEP, or TCEP + TOB B) Macroscopic moduli of biofilms treated with PBS, Tob, TCEP, or TCEP + TOB at 1 Hz. Statistical significance was determined with a two-tailed Student's *t*-test *p < 0.05, **p < 0.001 compared to the PBS control. C) Microscopic complex viscosity of biofilms at 1 Hz treated with PBS, Tob, TCEP, or TCEP + TOB. D) Microscopic storage (G′, solid) and loss (G″, dashed) moduli of treated biofilms showing the biofilm component (green) and the water component (blue) within the biofilm as determined with Gaussian Mixture Modeling. (For interpretation of the references to colour in this figure legend, the reader is referred to the Web version of this article.)

Biofilms were treated with tobramycin, an aminoglycoside antibiotic commonly used to treat Gram-negative pathogens [47,48]. A 5-log reduction in bacterial viability was achieved at 1000 μg/mL tobramycin (Fig. 4A). In agreement with previous work, the viscoelastic properties of biofilms were not significantly changed with tobramycin exposure (Fig. 4B) though there was a slight increase in moduli at 10 μg/mL tobramycin (Tob) [27]. There were no significant changes in biofilm viscoelastic properties at 1000 μg/mL Tob despite the 5-log reduction in bacterial viability. To disrupt the biofilm architecture and potentially improve antibiotic action, biofilms were treated with a reducing agent, TCEP (10 mM) to degrade disulfide bonds between and within mucin chains as well as other proteins within the biofilm matrix [49,50]. Concomitant exposure of biofilms to TCEP and tobramycin was evaluated for bactericidal action and biofilm disruption. There was no synergistic bactericidal action with TCEP (10 mM) and Tob (1000 μg/mL) treatment (Fig. 5). However, biofilm macroscopic moduli decreased (Fig. 5B), and microscale heterogeneity increased, indicating improved biofilm disruption (Fig. 5C). The Kolmogorov-Smirnov test determined that all η* distributions were significantly different (p < 0.05, Table 1). The non-Gaussian parameter, κ, is used to quantify heterogeneity of a sample, with greater values indicating a more heterogeneous distribution [10,40]. With Tob treatment, biofilms become more homogeneous (Table 1) while TCEP increased microscale heterogeneity. Combination treatment increased heterogeneity compared to the untreated biofilm control, increasing κ by ∼2.5X. Thus, simultaneously degrading the biofilm architecture and exerting antibiotic action resulted in superior biofilm disruption compared to Tob treatment alone.

**Table 1:** Statistical analysis of tobramycin and TCEP treatment of *P. aeruginosa* biofilms. The Kolmogorov-Smirnov test was applied to each ensemble of η* values at 1 Hz, comparing each treatment to the PBS-treated control biofilm. The non-Gaussian parameter, κ, was determined using each sample ensemble η* kurtosis at 1 Hz.

Treatment	p-Value	κ
PBS	NA	2.92
Tob 1000 μg/mL	2.1024e-31	0.49
TCEP 10 mM	5.3277e-117	25.42
TCEP + Tob	3.7779e-69	7.49

**Fig. 6:**
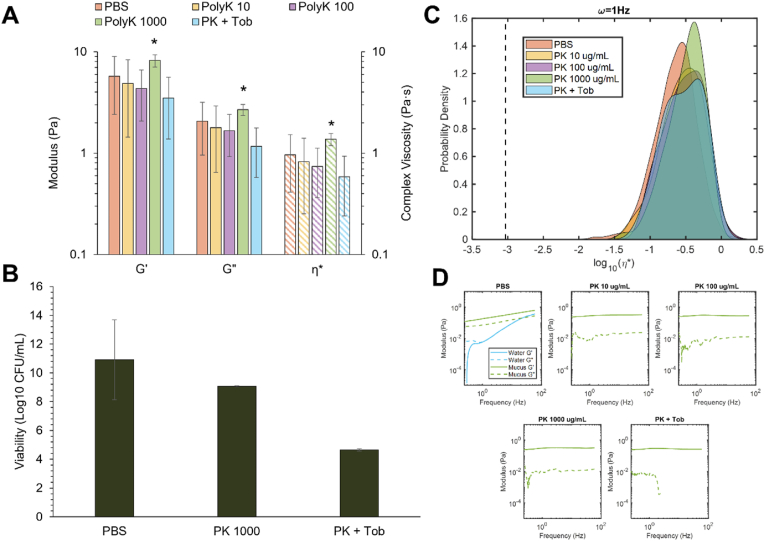
Viscoelastic properties of *P. aeruginosa* biofilms after treatment with tobramycin and PK. A) Macroscopic moduli of biofilms treated with PBS, PK, or PK + Tob. B) Viability of biofilms treated with PBS, PK or PK + Tob, each at 1000 μg/mL. C) Microscopic complex viscosity of biofilms treated with PBS, PK, or PK + Tob. D) Microscopic storage and loss moduli of biofilms treated with PBS, PK, or PK + Tob, each at 1000 μg/mL. Statistical significance was determined using a two-tailed Student's *t*-test *p < 0.05 compared to the PBS control.

Other methods of biofilm disruption were investigated for their effect on antibiotic susceptibility, specifically biopolymers due to their biocompatibility and the potential for preparation as an inhaled therapeutic. Two biopolymers, >30 kDa poly-l-lysine (PK) and 80–110 kDa hyaluronic acid (HA) were evaluated to represent a cationic and anionic biopolymer system, respectively. In keeping with previous results [27], biofilm treatment with a cationic polymer (PK) resulted in slight biofilm contraction and increased G’, G”, and η* (Fig. 6) though increases in macrorheology were not statistically significant until 1000 μg/mL PK. On the microrheological scale, η* values significantly increased with treatment (Fig. 6C and Table 2), with the water signal disappearing at even the lowest concentration of PK (Fig. 6D). Treatment with all doses of PK decreased κ, indicative of biofilm homogenization. The consistency of microrheology values with every tested dose of PK indicate that even small concentrations of a cationic biopolymer are sufficient to increase biofilm elasticity.

**Table 2:** Statistical analysis of tobramycin and PK treatment of *P. aeruginosa* biofilms. The Kolmogorov-Smirnov test was applied to each ensemble of η* values at 1 Hz, comparing each treatment to the PBS-treated control biofilm. The non-Gaussian parameter, κ, was determined using each sample ensemble η* kurtosis at 1 Hz.

Treatment	p-Value	κ
PK 10 μg/mL	0.0073	0.30
PK 100 μg/mL	9.3059e-06	0.50
PK 1000 μg/mL	3.1876e-11	−0.14
PK + Tob	8.5300e-06	−0.13

**Fig. 7:**
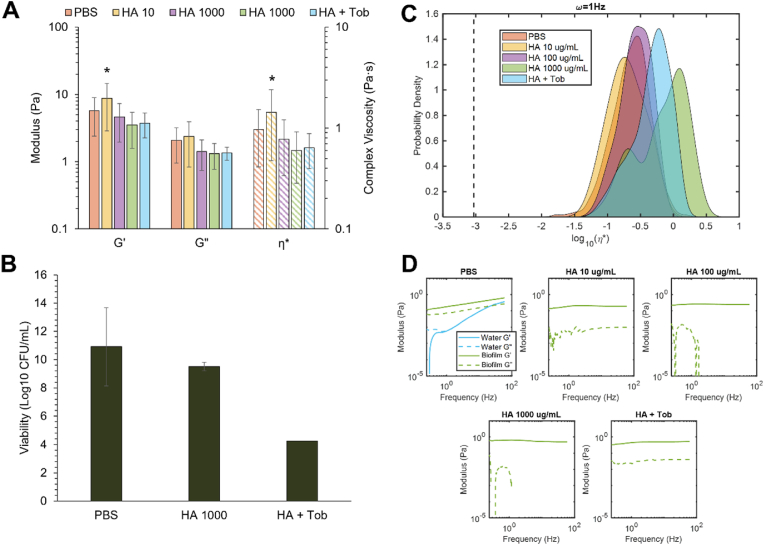
Viscoelastic properties of *P. aeruginosa* biofilms after treatment with tobramycin at 1000 μg/mL HA at 10, 100 or 1000 μg/mL. A) Macroscopic moduli of biofilms treated with PBS, HA, or HA + Tob. B) Viability of biofilms treated with PBS, HA, or HA + Tob, each at 1000 μg/mL. C) Microscopic complex viscosity of biofilms treated with PBS, HA, or HA + Tob. D) Microscopic storage and loss moduli of biofilms treated with PBS, HA, or HA + Tob, each at 1000 μg/mL. Statistical significance was determined using a two-tailed Student's *t*-test *p < 0.05 compared to the PBS control.

In contrast, moderate decreases in viscoelastic moduli were measured after treatment with HA (Fig. 7). Microscale homogeneity increased with HA treatment (Table 3) and the appearance of a lower η* component was observed at 1000 μg/mL HA (Fig. 7C). Additionally, the water signal disappeared with HA treatment, similar to PK (Fig. 7D). Alone, neither biopolymer significantly decreased biofilm viability (Fig. 7B). However, the combinations of tobramycin with PK or HA at 1000 μg/mL each resulted in a greater than 6-log reduction in bacterial viability (Fig. 6, Fig. 7B) and trending reductions in macroscopic moduli (Fig. 6, Fig. 7A).

**Table 3:** Statistical analysis of tobramycin and HA treatment of *P. aeruginosa* biofilms. The Kolmogorov-Smirnov test was applied to each ensemble of η* values at 1 Hz, comparing each treatment to the PBS-treated control biofilm. The non-Gaussian parameter, κ, was determined using each sample ensemble η* kurtosis at 1 Hz.

Treatment	p-Value	κ
HA 10 μg/mL	0.0695	−0.14
HA 100 μg/mL	0.0422	−0.18
HA 1000 μg/mL	4.6961e-105	−0.12
HA + Tob	7.8468e-65	−0.17

**Fig. 8:**
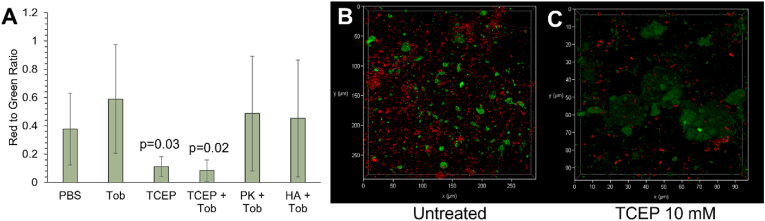
Microscopy imaging of *P. aeruginosa* biofilm in 4% mucus treated with tobramycin (1000 μg/mL) alone, TCEP (10 mM) alone, or tobramycin in combination with TCEP, PK, or HA, each at 1000 μg/mL. (A) Red to green pixel ratios as quantified with Matlab scripts using green vs red pixels. Data is presented as the average ± standard deviation of all pixels in ten images from varying locations in the biofilm. Statistical significance determined using a two-tailed Student's t-test compared to the PBS-treated control for n = 10 images per condition. (B) Representative image of untreated biofilm. C) Representative image of TCEP treated biofilm Green: BacLight Green (live bacteria). Red: propidium iodide (DNA). . (For interpretation of the references to colour in this figure legend, the reader is referred to the Web version of this article.)

Biofilm composition was also assessed with confocal microscopy to visualize the biofilm architecture with combination treatment. Propidium iodide stains DNA in the environment and in compromised cells. Because biofilms inherently contain extracellular DNA, the staining of DNA in the matrix artificially alters the ratio of red to green pixel ratios i.e., amount of DNA to live bacteria. Thus, we quantified red and green pixels from multiple images of biofilms to determine the effects of each treatment on the amount of DNA within the biofilm, both from extracellular DNA and dead bacteria, and the number of live bacteria. All treatments except TCEP and TCEP + Tob exhibited similar staining with comparable ratios of red to green pixels (Fig. 8). Uniquely, treatment with TCEP resulted in an increased ratio of live bacteria to DNA, which suggests that TCEP treatment fundamentally altered the detection of DNA in the biofilm, which has been observed in the literature [49]. We originally hypothesized that TCEP, which is a reducing agent, would reduce disulfide bonds between mucin chains within the mucus mesh. However, our data suggest that the decreased ratio of DNA to live bacteria is due to DNA degradation by TCEP. Thus, it is possible that TCEP decreases biofilm G’, G”, and η* by degrading DNA rather than disrupting the disulfide interactions in the mucus and biofilm mesh network.

### 4.3 Mucociliary transport of *P. aeruginosa* biofilms is improved with combination treatment

**Fig. 9:**
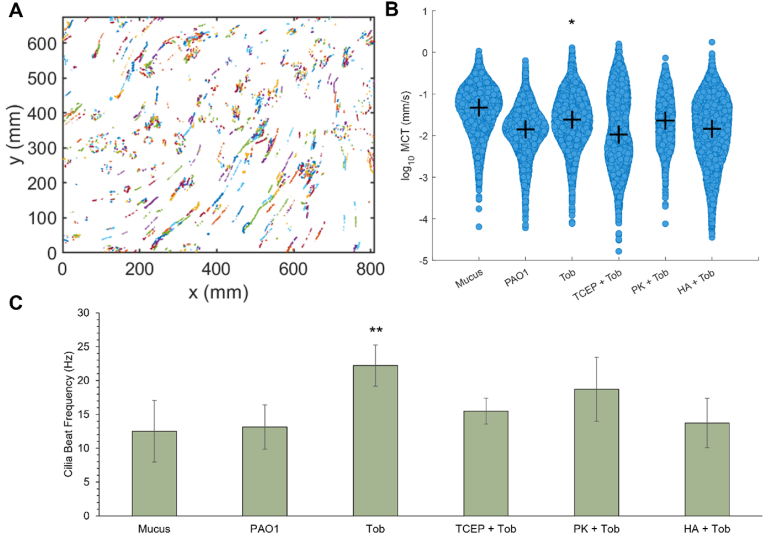
Racetrack transport of mucus or biofilms. A) Representative image of particle transport. B) Mucociliary transport rates of 4% mucus or *P. aeruginosa* biofilms in ALI cultures. Black plus signs represent the median MCT. Statistical significance was determined with a Kolmogorov-Smirnov test * p < 0.05 compared to PAO1. C) Average cilia beat frequency measurements for each treatment ± standard deviation. Statistical significance was determined by a two-tailed Student's *t*-test **p < 0.001 compared to PAO1. Data is representative of every tracked bead from biological duplicate exposures in cross exposed ALI cultures.

Previous work by Gloag and colleagues utilized viscoelastic moduli to quantify mucociliary clearance indices (MCI) and cough clearance indices (CCI) of *P. aeruginosa* biofilms and determined that the development of partially elastic behavior increased MCI and CCI [17]. To build upon this work, we evaluated mucociliary transport (MCT) of mucus-grown *P. aeruginosa* biofilms in well differentiated HBE cultures at the air-liquid interface (ALI) in a mucociliary transport device (MCTD) [42,51]. A representative image of tracked particle transport is shown in Fig. 9A. Uninfected mucus was evaluated as a benchmark for normal MCT (Fig. 9B). The untreated PAO1 biofilm moderately exhibited worse transport compared to 4% HBE mucus (p = 0.0529) despite normal CBF (Fig. 9C). The biofilm remained mostly intact for the duration of the experiment, indicating that MCT alone is insufficient to compromise the mechanical stability of the biofilm. The transport of treated biofilms was in agreement with macro- and microrheology measurements wherein greater viscoelastic properties was associated with slower MCT rates. Treatment with Tob alone statistically (p = 0.0205) increased MCT compared to the untreated biofilm as determined using the Kolmogorov-Smirnoff test. Treatment with PK + Tob or HA + Tob increased MCT to the value measured with Tob treatment but were not statistically distinct from the untreated biofilm. The bimodal distribution of the TCEP + Tob biofilm is unique to this treatment and could be due to DNA degradation, resulting in compromised biofilm architecture. No treatment raised MCT rate to that of uninfected mucus. Notably, CBF in the Tob-treated cultures was statistically greater than when cultures were exposed to uninfected mucus (p = 0.007) or the untreated biofilm (p = 0.0006), as determined by a two-tailed Student's *t*-test. The greater CBF observed in these cultures may contribute to increased MCT measured. While tobramycin nebulized solution has been shown to have no effect on CBF [52], it is possible that the injection of tobramycin treatment into a PAO1 biofilm initiates a signaling cascade that increases CBF.

## 5 Discussion

It is well established that the viscoelastic properties of mucus are responsible for its biological function and also that mucus concentrations become pathological in MOPD, altering viscoelastic properties, and compromising normal biological function [2,3,30]. Similarly, the viscoelastic properties of biofilms are critical for colony defense and response to mechanical and chemical challenges [16]. We posit that diseased airways are an ideal environment for chronic biofilm infections due to contributions from pathological mucus, compromised mucociliary transport, and the mechanical robustness observed in biofilms grown in mucus. We have previously shown that mucus concentration is a predominant contributor to the viscoelastic properties of biofilms, with biofilms grown in 5% mucus exhibiting greater viscoelastic moduli and resistance to enzymatic disruption compared to biofilms grown in 2% mucus [10]. In this present study, we evaluated the antibiotic efficacy of tobramycin as a function of biofilm physical disruption through treatment with the reducing agent TCEP, a cationic biopolymer poly-l-lysine, or an anionic biopolymer hyaluronic acid. Similar to mucus, biofilms were dominated by the elastic modulus (Fig. 1) and exhibited microscale heterogeneity due to the extracellular polymeric substances that comprise the biofilm matrix (Fig. 2) [9]. This heterogeneity was probed with a variety of beads of varied sizes and surface chemistries. The large, muco-inert bead PEG 1 μm was significantly excluded from the biofilm and associated with a more water-like component of the matrix, resulting in a bimodal distribution of measured η* values. In contrast, carboxylated beads and 0.5 μm PEG beads were sufficiently intercalated within the biofilm matrix and probed similar η* values (Fig. 2).

The 1-directional migration assay (1-DMA) biofilm nanorheology experiments demonstrated that 20 nm carboxylated beads were able to effectively penetrate and diffuse through the biofilm (Fig. 3). Indeed, the 20 nm beads measured the viscosity of water, indicating they flowed through the biofilm mesh network with the low viscosity fluid and thus the pore size of the biofilm matrix is greater than 20 nm [3,53]. The PK probe exhibited heterogeneous diffusion through the biofilm, which may be due to ionic interactions between the cationic polymer and the EPS components of the biofilm, which has previously been observed with chitosan oligosaccharides [27]. Uniquely, the smallest probes, the dextrans, diffused through the biofilm significantly faster than they diffused in water (Fig. 3). These data suggest that the 1–10 nm diameter dextran probes may be actively transported through the biofilm. Previous studies have shown that diffusion is the dominant form of transport of solutes within a biofilm even in the presence of advective flow [54]. However, in our analysis there is no source of fluid flow other than diffusion and any motility from the bacterial cells themselves. Indeed, fluorescence recovery after photobleaching (FRAP) studies of biofilms have indicated that within biofilms intrinsic and artifactual bacterial motion can occur [44]. While Waharte and colleagues determined that dextran diffusion within their biofilms was always slower compared to water [44], our dextrans diffused more rapidly in our biofilms compared to water. Thus, transport through biofilms may be significantly impacted by species and growth conditions, similar to the composition of the biofilm matrix [9].

The ultimate impact of transport through the biofilm on therapeutic efficacy is not yet well understood. Previous work has shown that the penetration of vancomycin into a *Staphylococcus aureus* was slowed compared to diffusion in water and authors suggested that slowed exposure facilitated a defense response from interior bacteria [55]. Our work suggests that agents with a 1–10 nm radius may be actively transported through our mucus-grown *P. aeruginosa* biofilms, but that >10 nm radius agents may diffuse with water through the biofilm matrix (Fig. 3). This phenomenon has an upper limit dependent on the biofilm mesh size, and the PTMR studies show that 500 nm carboxylated microspheres are larger than the mesh and intercalate within the biofilm (Fig. 2). We hypothesize that rapid diffusion through the biofilm would be detrimental for antibacterial efficacy as the agent will have insufficient time to exert action. However, further work is needed to understand the relationship between antibacterial size, biofilm penetration, and bacterial susceptibility to optimize biofilm penetration and antibacterial residence time. We also posit that surface chemistry will significantly contribute to interactions with the polymer network of the biofilm [43]. Future work will investigate a broader range of nanoparticle diameters and surface chemistries for diffusion through the biofilm mesh [43].

Biofilm treatments inherently alter viability and viscoelastic properties [10,13,56]. Ideally, both factors will be decreased to facilitate improved eradication and clearance, respectively. Treatment with tobramycin alone does not significantly alter the viscoelastic properties of biofilms, despite decreasing biofilm viability (Fig. 4). Further, MCT is moderately improved with tobramycin treatment (Fig. 9), but improved biofilm disruption is needed to facilitate complete biofilm eradication and clearance. To this end, biofilms were disrupted with TCEP, which has been shown to exert significant mucolytic action in pathological mucus due to disulfide bond reduction [57]. Our data indicate that TCEP also degrades DNA (Fig. 8). While TCEP did not impart synergistic bactericidal action with tobramycin, macroscopic and microscopic moduli of TCEP + Tob biofilms were reduced compared to tobramycin alone (Fig. 5). However, TCEP + Tob treatment was associated with decreased median MCT in ALI cultures (Fig. 9). The decrease in the viscoelastic properties of TCEP-treated biofilms may be due to decreased DNA in the biofilm (Fig. 7), but this effect was insufficient to improve MCT. This treatment was the only treatment type to cause a bimodal distribution of MCT in the biofilm. It is possible that compromised structural integrity via TCEP and bacterial death via tobramycin allowed for MCT to break the biofilm into a faster moving component and a slower moving component. To our knowledge, this is the first evaluation of MCT of mucus-grown biofilms and warrants further future study. Uniquely, both PK and HA treatment with tobramycin resulted in a >6-log reduction in biofilm viability, significant decreases in G′, G″, and η* (Fig. 6), and improved MCT (Fig. 9), though it was not restored to the rate of uninfected mucus. Thus, the most complete antibiofilm strategy was the combination of a biopolymer with tobramycin, which resulted in improved bactericidal action, decreased in G’, G”, and η*, and increased MCT. Distinctively, both PK and HA performed similarly, indicating that the polymer charge does not significantly influence biofilm disruption capability.

Rubenstein and Semenov have written extensively about the dynamics of entangled solutions of associating polymers, which may explain some of the trends observed in biopolymer-treated biofilms [58,59]. If a biofilm may be considered an entangled associating polymer complex, the interactions between associating groups (stickers) are controlled by the making and breaking of reversible bonds [58]. At a high degree of association, a sticker that breaks a bond is most likely to return to its original partner rather than to make a new bond [58]. At high concentrations, polymers are entangled and their dynamics are described by a sticky reptation model [58,59]. When biofilms are treated with PK or HA, the concentration of polymers in the biofilm increases, providing new bonding sites for the stickers that alters the mesh structure and facilitates flow. Alterations of the biofilm mesh and improved low-viscosity solvent flow through that mesh together may improve the diffusion of tobramycin and explain the increased antibiotic efficacy observed in combination treated biofilms, which has been observed in previous studies that degrade biofilms with DNase and treat with antibiotics [24,25].

In conclusion, we have begun to characterize the relationship between the viscoelastic properties of biofilms and viability with different treatment modalities. Simple bactericidal action is insufficient for treating chronic respiratory infections in muco-obstructive pulmonary diseases where MCC is compromised, and pathological mucus facilitates the formation of robust biofilms that are resistant to both mechanical and chemical challenges. Thus, antibiofilm strategies must both target the biofilm architecture and exert bactericidal action in order to effectively eradicate chronic infection from the airway. We have demonstrated that biofilm disruption with biopolymer treatment and concomitant treatment with tobramycin results in decreased biofilm viability, reduced viscoelastic moduli and complex viscosity, and improved mucociliary transport. Further either cationic or anionic biopolymers may be used to potentiate the action of tobramycin and facilitate biofilm eradication which increases the library of potential formulations for nebulization. Immediate next steps include preparations of hyaluronic acid and tobramycin for nebulization, and future work will evaluate this treatment strategy in chronically infected in vivo models.

## Funding

Funding was provided by the 10.13039/100000897Cystic Fibrosis Foundation (BOUCHE19R0, HILL19G0, HILL20Y2-OUT, ROUILL22F0).

## CRediT authorship contribution statement

**Kaitlyn R. Rouillard:** Conceptualization, Data curation, Formal analysis, Funding acquisition, Investigation, writing. **Matthew R. Markovetz:** Formal analysis, Methodology, Writing – review & editing. **William J. Kissner:** Resources, Writing – review & editing. **William L. Boone:** Methodology. **Lucas M. Plott:** Data curation. **David B. Hill:** Supervision, Funding acquisition, Methodology, Validation, Writing – review & editing.

## Declaration of competing interest

The authors declare that they have no known competing financial interests or personal relationships that could have appeared to influence the work reported in this paper.

## Data Availability

Data will be made available on request.
